# Supplementary material for: PROTACs and Building Blocks: The 2D Chemical Space in Very Early Drug Discovery
Source: Molecules. 2021 Jan 28;26(3):672. doi: 10.3390/molecules26030672 (PMC7865272; doi:10.3390/molecules26030672)
Supplement: Supplementary file 1 [file molecules-26-00672-s001.pdf]

PROTACs and building blocks: the 2D chemical space in very early drug discovery

Giulia Caron, Diego Garcia Jimenez and Giuseppe Ermondi*

Molecular Biotechnology and Health Sciences Dept., University of Torino, Via Quarello, 15, 10135 Torino, Italy

E-mail: giuseppe.ermondi@unito.it

Supporting Material

Tables

Table S1. Descriptors, classification and definition

Table S2. Complete spectra of the E3 ligases and their ligands, used for degrader formation

Table S3. Complete spectra of the proteins of interest and their warheads, used for degrader formation

Table S4. PCA variance

Table S5. E3 ligand data

Table S6. Warhead data

Table S7. Linker data

Table S8. PROTAC data

Table S9. bRo5 data

Table S10. Pearson's Correlation Coefficients and p values for E3 ligands descriptors correlations

Table S11. Pearson's Correlation Coefficients and p values for warhead descriptors correlations

Table S12. Pearson's Correlation Coefficients and p values for linkers descriptors correlations

Table S13. Pearson's Correlation Coefficients and p values for PROTACs descriptors correlations

Table S14. Descriptors used in the paper and the additional count-base descriptors

Table S15. Additional descriptors correlated with hydrophobicity descriptors used in the paper (Pearson coefficient)

Table S16. Additional descriptors correlated with polarity descriptors used in the paper (Pearson coefficient)

Table S17. Additional descriptors which are not significantly or few correlated with descriptors used in the paper (Pearson coefficient)

Figures

Figure S1. Databases overlap (PROTAC-DB in grey, PROTACpedia in yellow)

Figure S2. Investigated PROTAC chemical structures.

Figure S3. Box plots of the nonpolar descriptors for PROTACs and their building blocks with p-values obtained with an ANOVA analysis. A) MW, B) nC and C) NAR.nC and C) NAR.

Figure S4. Box plot of the polar descriptors for PROTACs and their building blocks obtained with p-values obtained with an ANOVA analysis. A) TPSA, B) nHAcc and C) nHDon

Figure S5. Box plot of the Kier's flexibility index (PHI) with p-values obtained with an ANOVA analysis.

Table S1. Descriptors, classification and definition

Descriptor name	Classification	Definition
**MW**	alvaDesc: Constitutional indices/Basic descriptors	*Molecular weight*
**nC**	alvaDesc: Constitutional indices/Basic descriptors	*Number of carbon atoms*
**NAR**	DataWarrior: Counts	*Number of aromatic rings*
**PHI**	alvaDesc: Topological indices/Path/walk indices	*Kier flexibility index*
**TPSA**	alvaDesc: Molecular properties/Basic descriptors	*Topological polar surface area using N,O, S, P polar contribution*
**nHAcc**	alvaDesc: Functional group count/Basic descriptors	*Number of acceptor atoms for H-bonds (N, O, F)*
**nHDon**	alvaDesc: Functional group count/Basic descriptors	*Number of donor atoms for H-bonds (N, O)*

Table S2. Complete spectra of the E3 ligases and their ligands, used for degrader formation.

FAMILY/CLASS	*E3 LIGASE GENE NAME*	*E3 LIGASE UNIPROT ID*	*Nº E3 LIGANDS (DEFINED NAME)*	*Nº E3 LIGANDS (UNDEFINED NAME)*	*TOTAL Nº OF DEGRADERS*
IAPs	**cIAP1/BIRC2**	Q13490	BESTATIN, MV1, LCL 161 DERIVATIVE, GDC-0152 (4)	3	7
	**cIAP2/BIRC3**	Q13489	LCL 161 DERIVATIVE, GDC-0152 (2)	0	2
	**IAP (unspecified)**	-	-	4	4
	**XIAP**	P98170	MV1, LCL 161 DERIVATIVE, GDC-0152 (3)	6	10
CRBN	**CRBN**	Q96SW2	POMALIDOMIDE, THALIDOMIDE, LENALIDOMIDE, S-LENALIDOMIDE, TD-106 (5)	4	9
bHLH	**AhR**	P35869	BETA-NAPHTHOFLAVONE, ALPHA-NAPHTHOFLAVONE, ITE (3)	0	3
DCAF	**DCAF15**	Q66K64	-	1	1
SWIB/MDM2	**MDM2**	Q00987	NUTLIN-3, RG7388 RG7112, NUTLIN-3A, NUTLIN-3B (5)	0	5
RING-C_RNF114	**RNF114**	Q9Y508	NIMBOLIDE (1)	0	1
RING-HC_RNF4	**RNF4**	P78317	CCW 16 (1)	0	1
VHL	**VHL**	P40337	VH032, VH298, VH101, VHL-1, VL285 (5)	22	27

Table S3. Complete spectra of the proteins of interest and their warheads, used for degrader formation.

FAMILY/CLASS	POI GENE NAME	POI UNIPROT ID	Nº WARHEADS (DEFINED NAME)	Nº WARHEADS (UNDEFINED NAME)	TOTAL Nº OF DEGRADERS
Nuclear hormone receptor NR3	AR	P10275	ENZALUTAMIDE, ARN-509, BICALUTAMIDE, DHT (4)	15	19
	ESR1/ESR2	P03372/ Q92731	RALOXIFENE, AFIMOXIFENE, TAMOXIFEN, BAZEDOXIFENE, LASOFOXIFENE, ESTRONE, ESTRADIOL, AZD9496 (8)	0	8
Bromodomain and extra-terminal domain	BRD2	P25440	JQ1, QCA276, OTX-015, HJB97, BETI-211, I-BET726 (6)	1	7
	BRD3	Q15059	JQ1, QCA276, OTX-015, HJB97, BETI-211, I-BET726 (6)	1	7
	BRD4	O60885	JQ1, QCA276, OTX-015, BI2536, GSK-39, ABBV-075, HJB97, BETI-211, I-BET726 (9)	4	13
Bromodomain-containing protein	BRD7	Q9NPI1	BI-7273, BI-9564, GSK-39 (3)	0	3
	BRD9	Q9H8M2	BI-7273, BI-9564, GSK-39 (3)	0	3
CDC2/CDKX	CDK1	P06493	TMX-2039, TMX-3013, TMX-3010, TMX-3012 (4)	0	4
	CDK2	P24941	AT-7519, FN-1501, TMX-2039, TMX-3013, TMX-3010, TMX-3012 (6)	0	6
	CDK4	P11802	PALBOCICLIB, ABEMACICLIB, RIBOCICLIB, TMX-2039, TMX-3013, TMX-3010, TMX-3012 (7)	0	7
	CDK5	Q00535	TMX-2039, TMX-3013, TMX-3010, TMX-3012 (4)	0	4
	CDK6	Q00534	PALBOCICLIB, ABEMACICLIB, RIBOCICLIB, TMX-2039, TMX-3013, TMX-3010, TMX-3012 (7)	0	7
	CDK7	P50613	TMX-2039, TMX-3010 (2)	0	2
	CDK8	P49336	JH-VIII-49 (1)	0	1
	CDK9	P50750	WOGONIN, AT-7519, FN-1501, SNS-032, NVP-2, TMX-2039, TMX-3010 (7)	1	8
Bcl-2	MCL1	Q07820	A-1210477 (1)	2	3
SWIB/MDM2	MDM2	Q00987	MI-1242, MI-1061, RG7112 (3)	0	3
Sirtuin	SIRT2	Q8IXJ6	-	0	1
Poly (ADP-Ribose) Polymerase	PARP1	P09874	NIRAPARIB, OLAPARIB, RUCAPARIB, VELIPARIB (4)	0	4
	PARP2	Q9UGN5	VELIPARIB (1)	0	1
	PARP3	Q9Y6F1	RUCAPARIB (1)	0	1
CRBN	CRBN	Q96SW2	POMALIDOMIDE, THALIDOMIDE (2)	0	2
PI3/PI4-kinase	PI3KALPHA	P42336	ZSTK474 (1)	0	1
Bcr-Abl	BCR-ABL	Q16189	BOSUTINIB, DASATINIB, IMATINIB, GNF-5, ASCIMINIB, PONATINIB, HG-7-85-01, ABL001 DERIVATIVE (8)	0	8
	BCR-ABL T315I	Q16189	ASCIMINIB, PONATINIB (2)	1	3
TEC	BTK	Q06187	DESPROPENOYLIBRUTINIB, SPEBRUTINIB, BOSUTINIB, CGI1746, RN486, IBRUTINIB (6)	6	12
Nuclear hormone receptor NR3	ESRRA	P11474	-	2	2
Insulin receptor	ALK	Q9UM73	CERITINIB, BRIGATINIB A, BRIGATINIB B, BRIGATINIB C, TAE684 (5)	0	5
	ALK G1202R	Q9UM73	BRIGATINIB B (1)	0	1
	IGF-1R	P08069	RBX10080307 (1)	1	2
Aurora	AURKA	O14965	TAE684, CERITINIB (2)	0	2
FAK	FAK	Q05397	DEFACTINIB, CERITINIB, TAE684 (3)	0	1
Fes/fps	FER	P16591	TAE684, CERITINIB (2)	0	1
S6 kinase	RPS6KA1	Q15418	TAE684, CERITINIB (2)	0	2
Histone deacetylase	HDAC1	Q13547	CI-994 (1)	0	1
	HDAC2	Q92769	CI-994 (1)	0	1
	HDAC3	O15379	CI-994 (1)	0	1
	HDAC6	Q9UBN7	NEXTURASTAT A (1)	1	2
RAF	BRAF V600E	P15056	VEMURAFENIB, BI882370 (2)	0	2
	BRAF	P15056	RIGOSERTIB (1)	0	1
BTB domain containing domain (BTBD)	BCL6	P41182	-	0	1
CSF-1/PDGF receptor	FLT3	P36888	QUIZARTINIB (1)	0	1

EGF receptor	EGFR	P00533	LAPATINIB, GEFITINIB, AFATINIB, B30, XTF-262, AZD-9291, JBJ-07-149, JBJ-04-125-02 (8)	2	10
	EGFR L858R/T790M	P00533	XTF-262 (1)	0	1
	HER2	P04626	LAPATINIB (1)	0	1
	EGFR L858R	P00533	GEFITINIB, XTF-262 (2)	2	2
Tyr protein kinase	C-Met	P08581	FORETINIB, PHA665752 (2)	0	2
AXL/UFO	MERTK	Q12866	FORETINIB (1)	0	1
MAP kinase	P38alpha	Q16539	FORETINIB, PH-797804 (2)	0	2
	P38delta	O15264	FORETINIB (1)	0	1
TKL Ser/Thr protein kinase	RIPK2	O43353	FORETINIB (1)	2	3
GCN5	GCN5	Q92830	GSK4027 (1)	0	1
	PCAF	Q92831	GSK4027, GSK4028 (2)	0	2
PI3/PI4-kinase	PI3Kalpha	P42336	ZSTK474 (1)	0	1
FKBP1	FKBP12	P68106	RAPAMYCIN (1)	1	2
FKBP1	FKBP12 F36V	P68106	AP1867<br>ORTHO-AP1867 (2)	0	2
MAP kinase	MEK1	Q02750	PD0325901 (1)	1	2
	MEK2	P36507	PD0325901(1)	1	2
STAT	STAT3	P40763	SI-109 (1)	4	5
Bcl-2	BCL2	P10415	ABT-263 (1)	2	3
	BCL-xL	Q07817	ABT-263, A-1155463 (2)	1	3
BTN/MOG	PD-L1	Q9NZQ7	BMS-1198, BMS-8, BMS-1233 (3)	0	3
Cytochrome P450	CYP1B1	Q16678	-	1	1
Eukaryotic initiation factor 4E	eIF4E	P06730	7-BNGMP, FBN7GMP, BN7GDP, 7-(P-CHLOROBENZYL) GDP, GMP, GDP (6)	0	6
Fatty-acid binding protein (FABP)	CRABP-I	P29762	ATRA (1)	0	1
	CRABP-II	P29373	ATRA (1)	0	1
PDE6D/unc-119	PDEdelta	O43924	DELTAZINONE 1 (1)	0	3
NR1	RAR (A, B, G)	P10276, P10826, P13631	CH55 (1)	0	1
JAK	JAK1	P23458	PYRIMIDINE 1, QUINOXALINE 2 (2)	0	2
	JAK2	O60674	PYRIMIDINE 1, QUINOXALINE 2 (2)	0	2
Bromodomain containing family	PBRM1	Q86U86	-	0	1
SNF2/RAD54 helicase	SMARCA2	P51531	-	0	1
	SMARCA4	P51532	-	0	1
Pelle	IRAK4	Q9NWZ3	PF-06650833 (1)	9	10
CDC5/Polo	PLK1	P53350	BI2536, AZD1775 (2)	0	2
AGC Ser/Thr protein kinase	SGK3	Q96BR1	290-R, 308-R (2)	0	2
WD repeat ESC	EED	O75530	EED226 (1)	1	2
RAC	AKT1	P31749	GDC-0068 (1)	0	1
	AKT2	P31751	GDC-0068 (1)	0	1
	AKT3	Q9Y243	GDC-0068 (1)	0	1
RAS	KRAS	P01116	-	2	2
	KRAS G12C	P01116	MRTX849 (1)	1	2
Tubulin	Tubulin (A,B,C)	Q71U36, P68363, P0DPH7	MMAE<br>COMBRETASTATIN A-4 (2)	0	2
Tau/MAP	Tau	P10636	18F-T807 (1)	0	1
ADRM1	Rpn13	Q16186	RA190 (1)	0	1
SRC	Src	P12931	PP2 (1)	0	1
Glutamine amidotransferase	NS3	Q9NWL6	TELAPREVIR (1)	0	1
CSF-1/PDGF receptor	c-KIT	P10721	SUNITINIB (1)	0	1
I-kappa-B kinase	TBK1	Q9UHD2	-	1	1
Non-receptor class 2	SHP2	Q06124	-	1	1
Synuclein	Alpha-syn	P37840	BTA-1 (1)	2	3
Insulin receptor	TrkA	P04629	ENTRECTINIB, GNF-8625 (2)	0	2
	TrkB	Q16620	ENTRECTINIB, GNF-8625 (2)	0	2
	TrkC	Q16832	ENTRECTINIB, GNF-8625 (2)	0	2

Table S4. PCA variance

	PC1	PC2	PC3	TOT
**E3 ligands**	59.12	25.01	9.37	93.5
**Linkers**	59.93	17.41	16.6	93.94
**Warheads**	56.94	24.23	11.76	92.93
**PROTACs**	59.21	17.39	12.46	89.06

Table S5. E3 ligand data

Name	Mean	Std.dev.	Maximum	Minimum
**MW**	462	111	728	240
**nC**	24	6	38	12
**NAR**	2	0.7	3	1
**PHI**	8	2	12	3
**nHDon**	3	1	5	0
**nHAcc**	8	2	11	2
**TPSA**	113	34	193	30

Table S6. Warhead data

Name	Mean	Std.dev.	Maximum	Minimum
**MW**	479	127	1035	240
**nC**	24	7	52	10
**NAR**	3	1	6	0
**PHI**	7	3	22	2
**nHDon**	3	2	8	0
**nHAcc**	8	3	21	2
**TPSA**	109	45	279	12

Table S7. Linker data

Name	Mean	Std.dev.	Maximum	Minimum
**MW**	215	83	556	16
**nC**	11	4	25	1
**NAR**	0	0.5	2	0
**PHI**	9	5	33	0
**nHDon**	1	1	5	0
**nHAcc**	4	2	13	0
**TPSA**	55	29	158	0

Table S8. PROTAC data

Name	Mean	Std.dev.	Maximum	Minimum
**MW**	984	174	1963	534
**nC**	50	9	96	28
**NAR**	4.7	1	9	1
**PHI**	18	5	42	7
**nHDon**	4	2	11	1
**nHAcc**	18	3	38	9
**TPSA**	242	44	460	115

Table S9: bRo5 data

Name	Mean	Std.dev.	Maximum	Minimum
**MW**	765.300	127.11998	1202.840	504.720
**nC**	39.327	8.22889	62	20
**NAR**	2.058	1.893	6	0
**PHI**	14.222	4.63463	33.567	7.329
**nHDon**	3.423	1.62512	8	0
**nHAcc**	13.288	3.46034	23	4
**TPSA**	177.451	44.98033	278.800	42.680

Table S10. Pearson's Correlation Coefficients and p values for E3 ligand descriptor correlations.

E2Ligands

**TPSA**	1.00						
**nHAcc**	0.85<br>*p < .001*	1.00					
**nHDon**	0.69<br>*p < .001*	0.63<br>*p < .001*	1.00				
**PHI**	0.50<br>*p < .001*	0.70<br>*p < .001*	0.39<br>*6.77E-04*	1.00			
**NAR**	0.11<br>*0.359*	0.065<br>0.590	-0.30<br>*1.23E-02*	0.28<br>*2.02E-02*	1.00		
**nC**	0.32<br>*5.95E-03*	0.55<br>*p < .001*	0.11<br>*3.83E-01*	0.89<br>*p < .001*	0.45<br>*p < .001*	1.00	
**MW**	0.52<br>*p < .001*	0.72<br>*p < .001*	0.23<br>*5.79E-02*	0.91<br>*p < .001*	0.47<br>*p < .001*	0.95<br>*p < .001*	1.00
	**TPSA**	**nHAcc**	**nHDon**	**PHI**	**NAR**	**nC**	**MW**

Table S11. Pearson's Correlation Coefficients and p values for warhead descriptor correlations

**TPSA**	1.00						
**nHAcc**	0.81<br>*p < .001*	1.00					
**nHDon**	0.77<br>*p < .001*	0.55<br>*p < .001*	1.00				
**PHI**	0.50<br>*p < .001*	0.68<br>*p < .001*	0.20<br>*p < .001*	1.00			
**NAR**	0.14<br>*1.75E-02*	0.22<br>*7.48E-02*	-0.05<br>*4.16E-01*	0.18<br>*1.57E-03*	1.00		
**nC**	0.24<br>*p < .001*	0.46<br>*p < .001*	-0.09<br>*1.07E-01*	0.83<br>*p < .001*	0.46<br>*p < .001*	1.00	
**MW**	0.59<br>*p < .001*	0.75<br>*p < .001*	0.23<br>*p < .001*	0.90<br>*p < .001*	0.39<br>*p < .001*	0.87<br>*p < .001*	1.00
	**TPSA**	**nHAcc**	**nHDon**	**PHI**	**NAR**	**nC**	**MW**

Table S12. Pearson's Correlation Coefficients and p values for linker descriptor correlations

**TPSA**	1.00						
**nHAcc**	0.93<br>*p < .001*	1.00					
**nHDon**	0.62<br>*p < .001*	0.43<br>*p < .001*	1.00				
**PHI**	0.54<br>*p < .001*	0.67<br>*p < .001*	0.13<br>*3.02E-04*	1.00			
**NAR**	0.30<br>*p < .001*	0.224<br>*p < .001*	0.03<br>*3.68E-01*	-0.24<br>*p < .001*	1.00		
**nC**	0.56<br>*p < .001*	0.65<br>*p < .001*	0.11<br>*2.75E-03*	0.68<br>*p < .001*	0.41<br>*p < .001*	1.00	
**MW**	0.78<br>*p < .001*	0.87<br>*p < .001*	0.24<br>*p < .001*	0.75<br>*p < .001*	0.37<br>*p < .001*	0.94<br>*p < .001*	1.00
	**TPSA**	**nHAcc**	**nHDon**	**PHI**	**NAR**	**nC**	**MW**

Table S13. Pearson's Correlation Coefficients and p values for PROTAC descriptor correlations.

**TPSA**	1.00						
**nHAcc**	0.76<br>*p < .001*	1.00					
**nHDon**	0.59<br>*p < .001*	0.30<br>*p < .001*	1.00				
**PHI**	0.62<br>*p < .001*	0.66<br>*p < .001*	0.33<br>*p < .001*	1.00			
**NAR**	2.58E-01<br>*p < .001*	0.071<br>*1.08E-03*	0.03<br>*1.14E-01*	0.16<br>*p < .001*	1.00		
**nC**	0.50<br>*p < .001*	0.52<br>*p < .001*	0.16<br>*p < .001*	0.83<br>*p < .001*	0.47<br>*p < .001*	1.00	
**MW**	0.68<br>*p < .001*	0.72<br>*p < .001*	0.24<br>*p < .001*	0.88<br>*p < .001*	0.38<br>*p < .001*	0.93<br>*p < .001*	1.00
	**TPSA**	**nHAcc**	**nHDon**	**PHI**	**NAR**	**nC**	**MW**

Figure S1. Databases overlap (PROTAC-DB in grey, PROTACpedia in yellow)

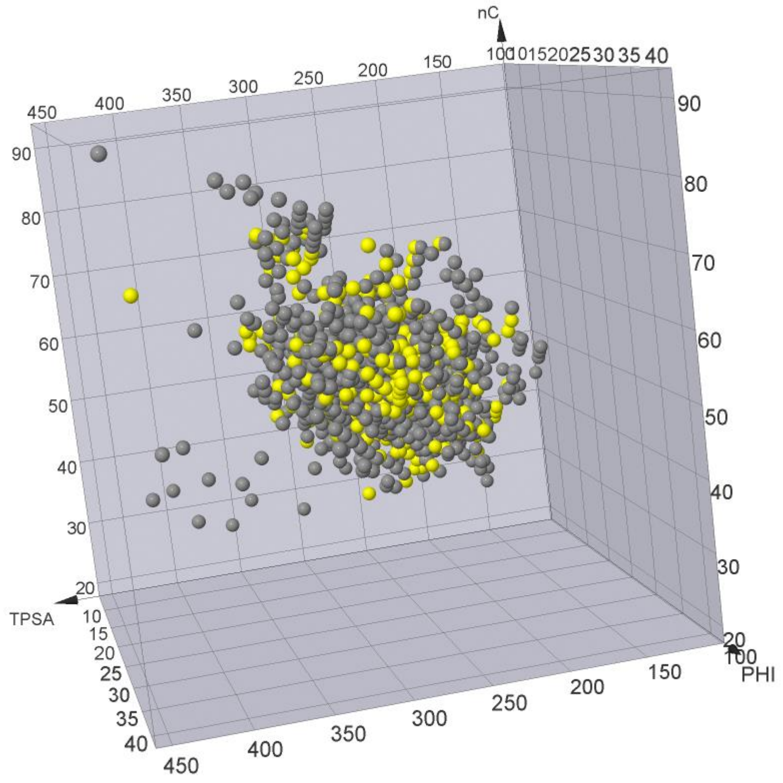

Figure S2. Investigated PROTAC chemical structures.

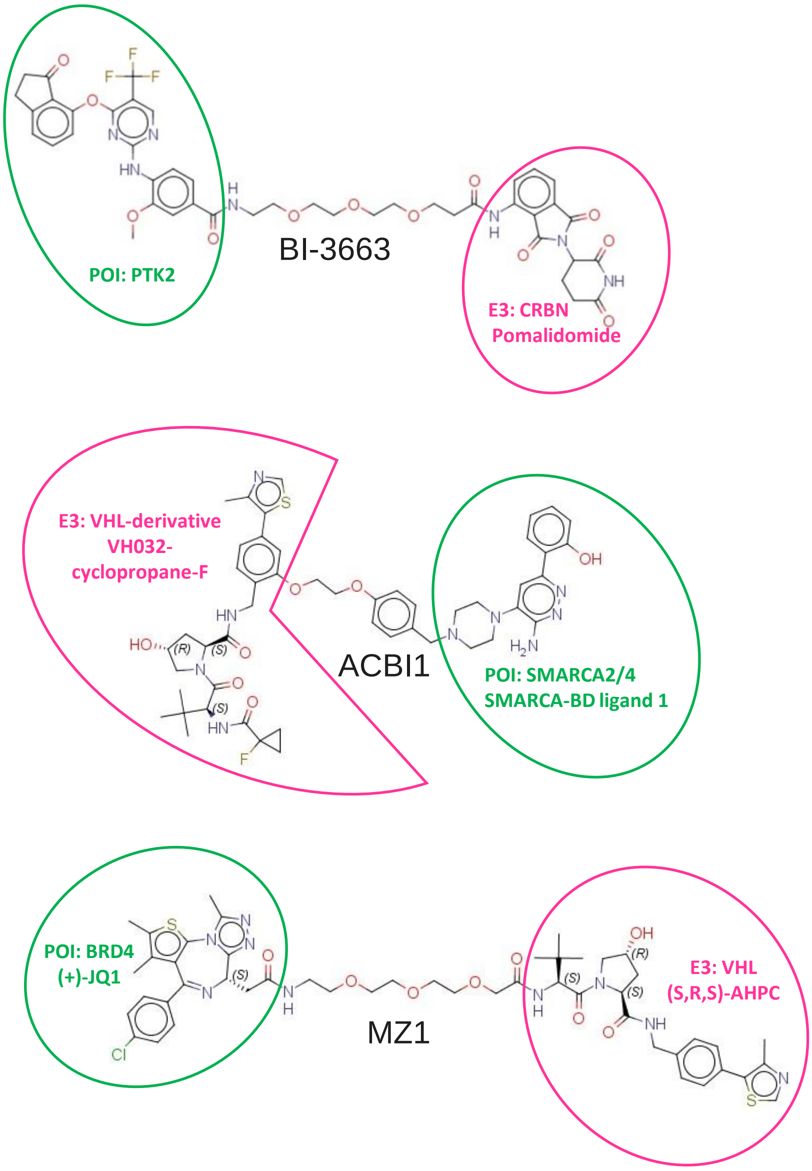

Figure S3. Box plots (same as Fig. 6) of the nonpolar descriptors for PROTACs and their building blocks with p-values obtained with ANOVA analysis. A) MW, B) nC and C) NAR.

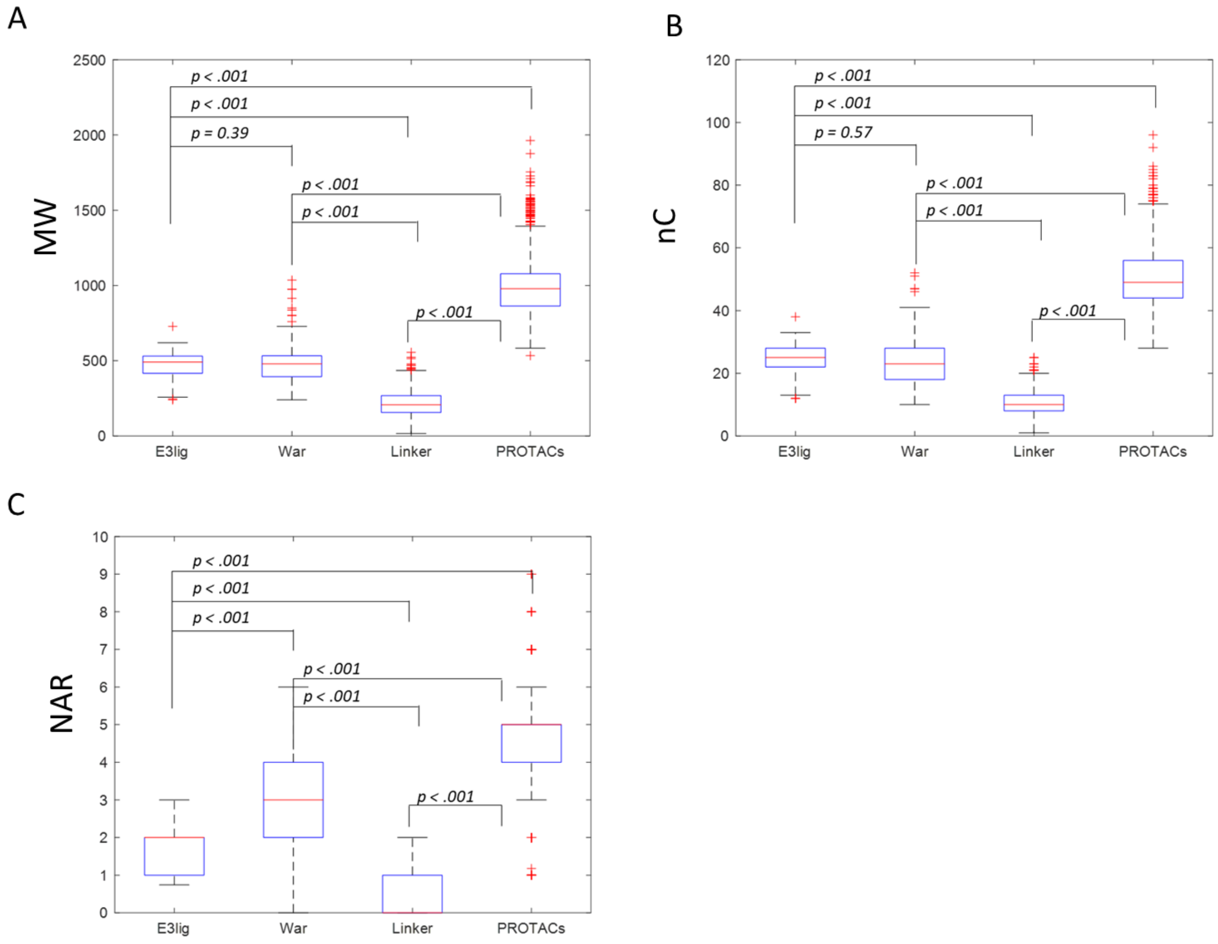

Figure S4. Box plot (same as Fig. 7) of the polar descriptors for PROTACs and their building blocks with p-values obtained with ANOVA analysis. A) TPSA, B) nHAcc and C) nHDon

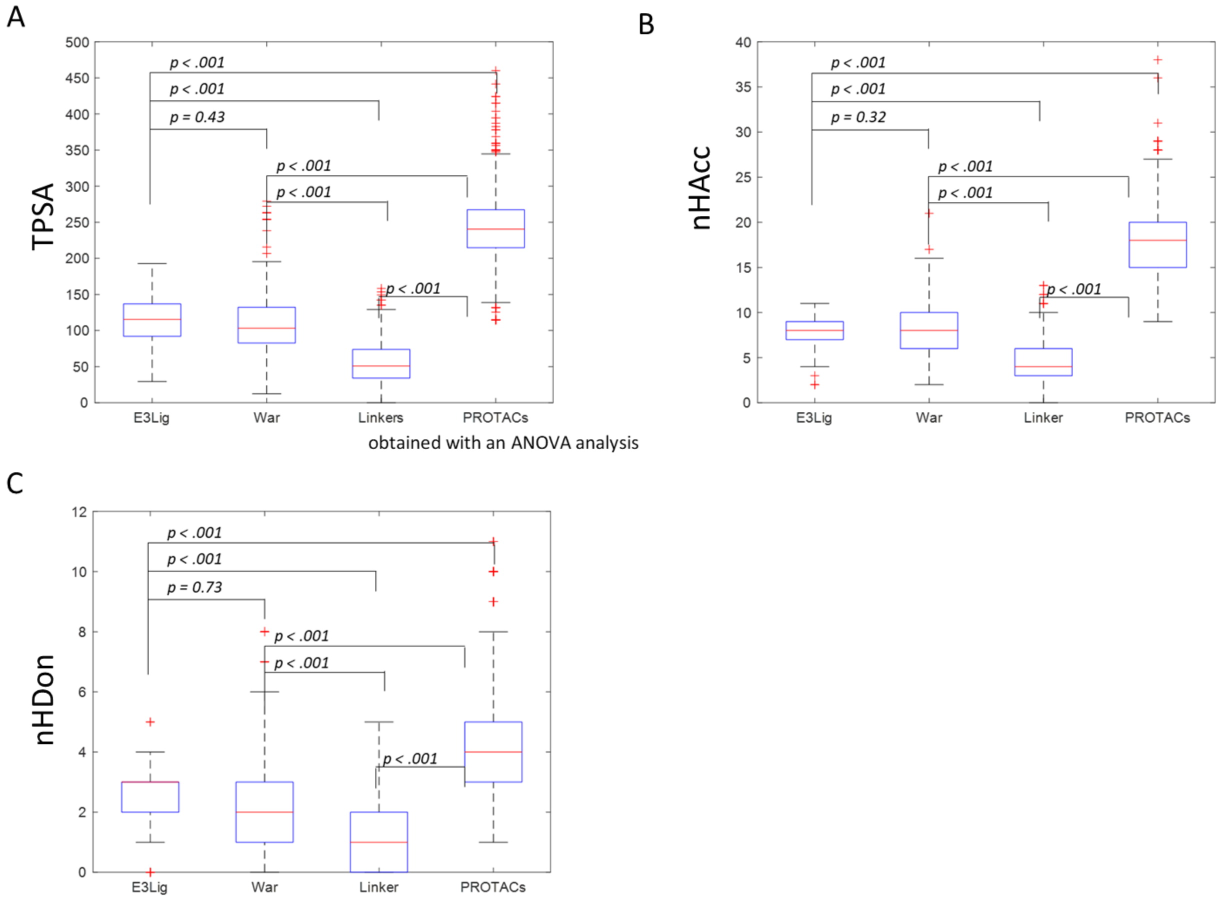

Figure S5. Box plot (same as Fig. 8) of the Kier's flexibility index (PHI) with p-values obtained with ANOVA analysis.

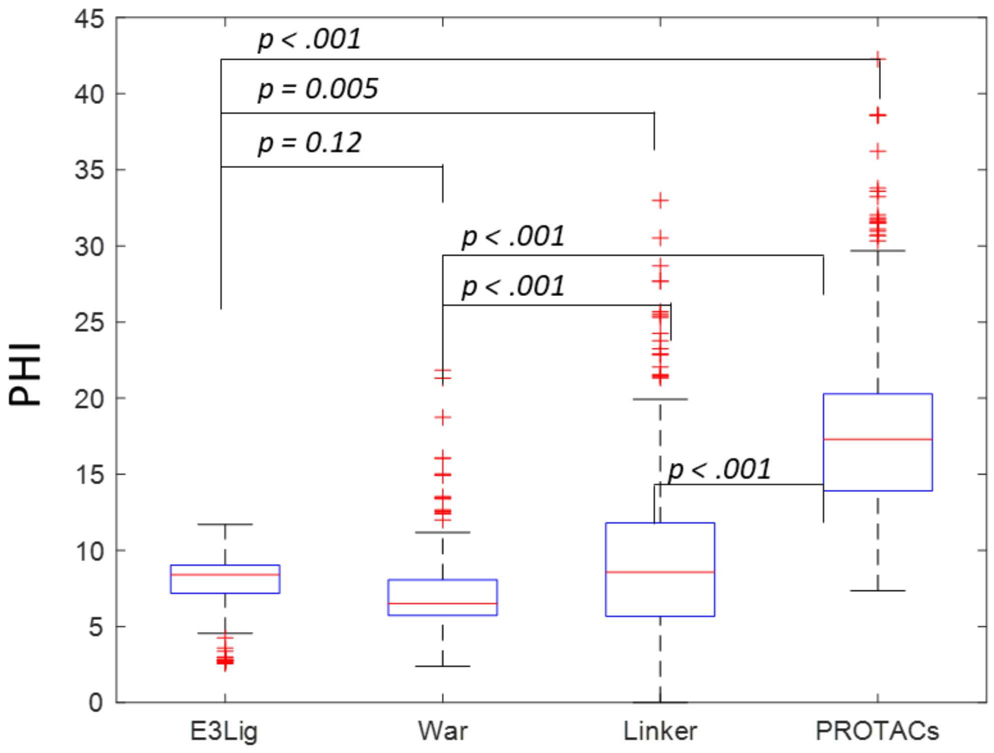

Descriptors selection

The choice of the 7 descriptors can be also justified on the basis of statistical criteria as described below. We calculated with the same computational tools (alvaDesc and DataWarrior) an additional set of 229 count-based descriptors (data in Table S14). All the descriptors with constant values, standard deviation less than 0.0001 and with a percentage of 0 values greater than 70% were discarded obtaining a final set of 46 descriptors. The correlations between the 7 descriptors used in the paper and the additional 46 were evaluated (Table S15-S17). Then, Pearson coefficients allowed to classify the additional 46 descriptors in either hydrophobic or polar (no additional flexibility determinant was found). For example, nO showed the highest correlation with TPSA and thus it was considered a polar descriptor since TPSA is a polar descriptor. Using this strategy, we identified 17 additional hydrophobic and 3 polar descriptors. As shown in Tables S15-S17, most of these latter are significantly correlated with the set of seven descriptors that we selected to represent polarity and hydrophobicity and thus do not increase the amount of information. The remaining few descriptors, not correlated with the original pool, do not have any clear physicochemical meaning and thus are not in line with our approach based on physicochemical properties. For example, the percentage of heavy atoms is difficult to relate to hydrophobicity/polarity. Overall, these findings support that the selected 7 descriptors are the most relevant to cover the physicochemical properties of the datasets.

Table S14. Descriptors used in the paper and the additional count-base descriptors

Name	Description	Block	Included
MW	molecular weight	Constitutional indices	1
nC	number of carbon atoms	Constitutional indices	1
Aromatic Rings	number of aromatic rings	Functional group counts	1
TPSA(Tot)	TPSA	Molecular Properties	1
nHAcc	number of acceptor atoms for H-bonds (N and O)	Functional group counts	1
nHDon	number of donor atoms for H-bonds (N and O)	Functional group counts	1
PHI	Kier flexibility index	Topological Index	1
AMW	average molecular weight	Constitutional indices	0
Sv	sum of atomic van der Waals volumes (scaled on Carbon atom)	Constitutional indices	0
Se	sum of atomic Sanderson electronegativities (scaled on Carbon atom)	Constitutional indices	0
Sp	sum of atomic polarizabilities (scaled on Carbon atom)	Constitutional indices	0
Si	sum of first ionization potentials (scaled on Carbon atom)	Constitutional indices	0
Mv	mean atomic van der Waals volume (scaled on Carbon atom)	Constitutional indices	0
Me	mean atomic Sanderson electronegativity (scaled on Carbon atom)	Constitutional indices	0
Mp	mean atomic polarizability (scaled on Carbon atom)	Constitutional indices	0
Mi	mean first ionization potential (scaled on Carbon atom)	Constitutional indices	0
GD	graph density	Constitutional indices	0
nAT	number of atoms	Constitutional indices	0
nSK	number of non-H atoms	Constitutional indices	0
nTA	number of terminal atoms	Constitutional indices	1
nBT	number of bonds	Constitutional indices	1
nBO	number of non-H bonds	Constitutional indices	1
nBM	number of multiple bonds	Constitutional indices	1
SCBO	sum of conventional bond orders (H-depleted)	Constitutional indices	1
RBN	number of rotatable bonds	Constitutional indices	1
RBF	rotatable bond fraction	Constitutional indices	1
nDB	number of double bonds	Constitutional indices	1
nTB	number of triple bonds	Constitutional indices	0
nAB	number of aromatic bonds	Constitutional indices	0
nH	number of Hydrogen atoms	Constitutional indices	0
nN	number of Nitrogen atoms	Constitutional indices	1
nO	number of Oxygen atoms	Constitutional indices	1
nP	number of Phosphorous atoms	Constitutional indices	0
nS	number of Sulfur atoms	Constitutional indices	0
nF	number of Fluorine atoms	Constitutional indices	0
nCL	number of Chlorine atoms	Constitutional indices	0
nBR	number of Bromine atoms	Constitutional indices	0
nI	number of Iodine atoms	Constitutional indices	0
nB	number of Boron atoms	Constitutional indices	0
nHM	number of heavy atoms	Constitutional indices	1
nHet	number of heteroatoms	Constitutional indices	1
nX	number of halogen atoms	Constitutional indices	1
H%	percentage of H atoms	Constitutional indices	0
C%	percentage of C atoms	Constitutional indices	1
N%	percentage of N atoms	Constitutional indices	1
O%	percentage of O atoms	Constitutional indices	1
X%	percentage of halogen atoms	Constitutional indices	1
nCsp3	number of sp3 hybridized Carbon atoms	Constitutional indices	1
nCsp2	number of sp2 hybridized Carbon atoms	Constitutional indices	1
nCsp	number of sp hybridized Carbon atoms	Constitutional indices	1
nStructures	number of disconnected structures	Constitutional indices	1
totalcharge	total charge	Constitutional indices	0
nCIC	number of rings (cyclomatic number)	Ring descriptors	1
nCIR	number of circuits	Ring descriptors	1
TRS	total ring size	Ring descriptors	1
Rperim	ring perimeter	Ring descriptors	1
Rbrid	ring bridge count	Ring descriptors	1
MCD	molecular cyclized degree	Ring descriptors	1
RFD	ring fusion density	Ring descriptors	1
RCI	ring complexity index	Ring descriptors	1
NRS	number of ring systems	Ring descriptors	1
NNRS	normalized number of ring systems	Ring descriptors	1
nR03	number of 3-membered rings	Ring descriptors	0
nR04	number of 4-membered rings	Ring descriptors	0
nR05	number of 5-membered rings	Ring descriptors	1
nR06	number of 6-membered rings	Ring descriptors	1
nR07	number of 7-membered rings	Ring descriptors	0
nR08	number of 8-membered rings	Ring descriptors	0
nR09	number of 9-membered rings	Ring descriptors	0

nR10	number of 10-membered rings	Ring descriptors	0
nR11	number of 11-membered rings	Ring descriptors	0
nR12	number of 12-membered rings	Ring descriptors	0
nBnz	number of benzene-like rings	Ring descriptors	0
ARR	aromatic ratio	Ring descriptors	0
D/Dtr03	distance/detour ring index of order 3	Ring descriptors	0
D/Dtr04	distance/detour ring index of order 4	Ring descriptors	0
D/Dtr05	distance/detour ring index of order 5	Ring descriptors	0
D/Dtr06	distance/detour ring index of order 6	Ring descriptors	0
D/Dtr07	distance/detour ring index of order 7	Ring descriptors	0
D/Dtr08	distance/detour ring index of order 8	Ring descriptors	0
D/Dtr09	distance/detour ring index of order 9	Ring descriptors	0
D/Dtr10	distance/detour ring index of order 10	Ring descriptors	0
D/Dtr11	distance/detour ring index of order 11	Ring descriptors	0
D/Dtr12	distance/detour ring index of order 12	Ring descriptors	0
nCp	number of terminal primary C(sp3)	Functional group counts	0
nCs	number of total secondary C(sp3)	Functional group counts	0
nCt	number of total tertiary C(sp3)	Functional group counts	0
nCq	number of total quaternary C(sp3)	Functional group counts	0
nCrs	number of ring secondary C(sp3)	Functional group counts	0
nCrt	number of ring tertiary C(sp3)	Functional group counts	0
nCrq	number of ring quaternary C(sp3)	Functional group counts	0
nCar	number of aromatic C(sp2)	Functional group counts	0
nCbH	number of unsubstituted benzene C(sp2)	Functional group counts	0
nCb-	number of substituted benzene C(sp2)	Functional group counts	0
nCconj	number of non-aromatic conjugated C(sp2)	Functional group counts	0
nR=Cp	number of terminal primary C(sp2)	Functional group counts	0
nR=Cs	number of aliphatic secondary C(sp2)	Functional group counts	0
nR=Ct	number of aliphatic tertiary C(sp2)	Functional group counts	0
n=C=	number of allenes groups	Functional group counts	0
nR#CH/X	number of terminal C(sp)	Functional group counts	0
nR#C-	number of non-terminal C(sp)	Functional group counts	0
nROCN	number of cyanates (aliphatic)	Functional group counts	0
nArOCN	number of cyanates (aromatic)	Functional group counts	0
nRNCO	number of isocyanates (aliphatic)	Functional group counts	0
nArNCO	number of isocyanates (aromatic)	Functional group counts	0
nRSCN	number of thiocyanates (aliphatic)	Functional group counts	0
nArSCN	number of thiocyanates (aromatic)	Functional group counts	0
nRNCS	number of isothiocyanates (aliphatic)	Functional group counts	0
nArNCS	number of isothiocyanates (aromatic)	Functional group counts	0
nRCOOH	number of carboxylic acids (aliphatic)	Functional group counts	0
nArCOOH	number of carboxylic acids (aromatic)	Functional group counts	0
nRCOOR	number of esters (aliphatic)	Functional group counts	0
nArCOOR	number of esters (aromatic)	Functional group counts	0
nRCONH2	number of primary amides (aliphatic)	Functional group counts	0
nArCONH2	number of primary amides (aromatic)	Functional group counts	0
nRCONHR	number of secondary amides (aliphatic)	Functional group counts	0
nArCONHR	number of secondary amides (aromatic)	Functional group counts	0
nRCONR2	number of tertiary amides (aliphatic)	Functional group counts	0
nArCONR2	number of tertiary amides (aromatic)	Functional group counts	0
nROCON	number of (thio-) carbamates (aliphatic)	Functional group counts	0
nArOCON	number of (thio-) carbamates (aromatic)	Functional group counts	0
nRCOX	number of acyl halogenides (aliphatic)	Functional group counts	0
nArCOX	number of acyl halogenides (aromatic)	Functional group counts	0
nRCSOH	number of thioacids (aliphatic)	Functional group counts	0
nArCSOH	number of thioacids (aromatic)	Functional group counts	0
nRCSSH	number of dithioacids (aliphatic)	Functional group counts	0
nArCSSH	number of dithioacids (aromatic)	Functional group counts	0
nRCOSR	number of thioesters (aliphatic)	Functional group counts	0
nArCOSR	number of thioesters (aromatic)	Functional group counts	0
nRCSSR	number of dithioesters (aliphatic)	Functional group counts	0
nArCSSR	number of dithioesters (aromatic)	Functional group counts	0
nRCHO	number of aldehydes (aliphatic)	Functional group counts	0
nArCHO	number of aldehydes (aromatic)	Functional group counts	0
nRCO	number of ketones (aliphatic)	Functional group counts	0
nArCO	number of ketones (aromatic)	Functional group counts	0
nCONN	number of urea (-thio) derivatives	Functional group counts	0
nC=O(O)2	number of carbonate (-thio) derivatives	Functional group counts	0
nN=C-N<	number of amidine derivatives	Functional group counts	0
nC(=N)N2	number of guanidine derivatives	Functional group counts	0
nRC=N	number of imines (aliphatic)	Functional group counts	0
nArC=N	number of imines (aromatic)	Functional group counts	0

nRCNO	number of oximes (aliphatic)	Functional group counts	0
nArCNO	number of oximes (aromatic)	Functional group counts	0
nRNH2	number of primary amines (aliphatic)	Functional group counts	0
nArNH2	number of primary amines (aromatic)	Functional group counts	0
nRNHR	number of secondary amines (aliphatic)	Functional group counts	0
nArNHR	number of secondary amines (aromatic)	Functional group counts	0
nRNR2	number of tertiary amines (aliphatic)	Functional group counts	0
nArNR2	number of tertiary amines (aromatic)	Functional group counts	0
nN-N	number of N hydrazines	Functional group counts	0
nN=N	number of N azo-derivatives	Functional group counts	0
nRCN	number of nitriles (aliphatic)	Functional group counts	0
nArCN	number of nitriles (aromatic)	Functional group counts	0
nN+	number of positively charged N	Functional group counts	0
nNq	number of quaternary N	Functional group counts	0
nRNHO	number of hydroxylamines (aliphatic)	Functional group counts	0
nArNHO	number of hydroxylamines (aromatic)	Functional group counts	0
nRNNOx	number of N-nitroso groups (aliphatic)	Functional group counts	0
nArNNOx	number of N-nitroso groups (aromatic)	Functional group counts	0
nRNO	number of nitroso groups (aliphatic)	Functional group counts	0
nArNO	number of nitroso groups (aromatic)	Functional group counts	0
nRNO2	number of nitro groups (aliphatic)	Functional group counts	0
nArNO2	number of nitro groups (aromatic)	Functional group counts	0
nN(CO)2	number of imides (-thio)	Functional group counts	0
nC=N-N<	number of hydrazones	Functional group counts	0
nROH	number of hydroxyl groups	Functional group counts	0
nArOH	number of aromatic hydroxyls	Functional group counts	0
nOHp	number of primary alcohols	Functional group counts	0
nOHs	number of secondary alcohols	Functional group counts	0
nOHt	number of tertiary alcohols	Functional group counts	0
nROR	number of ethers (aliphatic)	Functional group counts	1
nArOR	number of ethers (aromatic)	Functional group counts	0
nROX	number of hypohalogenides (aliphatic)	Functional group counts	0
nArOX	number of hypohalogenides (aromatic)	Functional group counts	0
nO(C=O)2	number of anhydrides (-thio)	Functional group counts	0
nH2O	number of water molecules	Functional group counts	0
nSH	number of thiols	Functional group counts	0
nC=S	number of thioketones	Functional group counts	0
nRSR	number of sulfides	Functional group counts	0
nRSSR	number of disulfides	Functional group counts	0
nSO	number of sulfoxides	Functional group counts	0
nS(=O)2	number of sulfones	Functional group counts	0
nSOH	number of sulfenic (thio-) acids	Functional group counts	0
nSOOH	number of sulfinic (thio-/dithio-) acids	Functional group counts	0
nSO2OH	number of sulfonic (thio-/dithio-) acids	Functional group counts	0
nSO3OH	number of sulfuric (thio-/dithio-) acids	Functional group counts	0
nSO2	number of sulfites (thio-/dithio-)	Functional group counts	0
nSO3	number of sulfonates (thio-/dithio-)	Functional group counts	0
nSO4	number of sulfates (thio-/dithio-)	Functional group counts	0
nSO2N	number of sulfonamides (thio-/dithio-)	Functional group counts	0
nPO3	number of phosphites/thiophosphites	Functional group counts	0
nPO4	number of phosphates/thiophosphates	Functional group counts	0
nPR3	number of phosphanes	Functional group counts	0
nP(=O)O2R	number of phosphonates (thio-)	Functional group counts	0
nP(=O)R3/nPR5	number of phosphoranes (thio-)	Functional group counts	0
nCH2RX	number of CH2RX	Functional group counts	0
nCHR2X	number of CHR2X	Functional group counts	0
nCR3X	number of CR3X	Functional group counts	0
nR=CHX	number of R=CHX	Functional group counts	0
nR=CRX	number of R=CRX	Functional group counts	0
nR#CX	number of R#CX	Functional group counts	0
nCHRX2	number of CHRX2	Functional group counts	0
nCR2X2	number of CR2X2	Functional group counts	0
nR=CX2	number of R=CX2	Functional group counts	0
nCRX3	number of CRX3	Functional group counts	0
nArX	number of X on aromatic ring	Functional group counts	0
nCXr	number of X on ring C(sp3)	Functional group counts	0
nCXr=	number of X on ring C(sp2)	Functional group counts	0
nCconjX	number of X on exo-conjugated C	Functional group counts	0
nAziridines	number of Aziridines	Functional group counts	0
nOxiranes	number of Oxiranes	Functional group counts	0
nThiranes	number of Thiranes	Functional group counts	0
nAzetidines	number of Azetidines	Functional group counts	0

nOxetanes	number of Oxetanes	Functional group counts	0
nThioethanes	number of Thioethanes	Functional group counts	0
nBeta-Lactams	number of Beta-Lactams	Functional group counts	0
nPyrrolidines	number of Pyrrolidines	Functional group counts	0
nOxolanes	number of Oxolanes	Functional group counts	0
ntH-Thiophenes	number of tetrahydro-thiophenes	Functional group counts	0
nPyrroles	number of Pyrroles	Functional group counts	0
nPyrazoles	number of Pyrazoles	Functional group counts	0
nImidazoles	number of Imidazoles	Functional group counts	0
nFuranes	number of Furanes	Functional group counts	0
nThiophenes	number of Thiophenes	Functional group counts	0
nOxazoles	number of Oxazoles	Functional group counts	0
nIsoxazoles	number of Isoxazoles	Functional group counts	0
nThiazoles	number of Thiazoles	Functional group counts	0
nIsothiazoles	number of Isothiazoles	Functional group counts	0
nTriazoles	number of Triazoles	Functional group counts	0
nPyridines	number of Pyridines	Functional group counts	0
nPyridazines	number of Pyridazines	Functional group counts	0
nPyrimidines	number of Pyrimidines	Functional group counts	0
nPyrazines	number of Pyrazines	Functional group counts	0
n135-Triazines	number of 1-3-5-Triazines	Functional group counts	0
n124-Triazines	number of 1-2-4-Triazines	Functional group counts	0
nHDon	number of donor atoms for H-bonds (N and O)	Functional group counts	1

Table S15. Additional descriptors correlated with hydrophobicity descriptors used in the paper (Pearson coefficient and p-values)

	nTA	nBT	nBO	SCBO	nCsp3	nCsp2	nCsp	nBM	RBN	nDB	nCIC	nCIR	TRS	Rperim	NRS	nR06	nHM
**MW**	0.89<br>*p<.001*	1.00<br>*p<.001*	1.00<br>*p<.001*	0.99<br>*p<.001*	0.84<br>*p<.001*	0.95<br>*p<.001*	0.93<br>*p<.001*	0.91<br>*p<.001*	0.88<br>*p<.001*	0.94<br>*p<.001*	0.91<br>*p<.001*	0.82<br>*p<.001*	0.91<br>*p<.001*	0.93<br>*p<.001*	0.93<br>*p<.001*	0.86<br>*p<.001*	1.00<br>*p<.001*
**nC**	0.88<br>*p<.001*	0.99<br>*p<.001*	0.99<br>*p<.001*	0.99<br>*p<.001*	0.84<br>*p<.001*	0.95<br>*p<.001*	0.94<br>*p<.001*	0.91<br>*p<.001*	0.88<br>*p<.001*	0.94<br>*p<.001*	0.92<br>*p<.001*	0.82<br>*p<.001*	0.92<br>*p<.001*	0.93<br>*p<.001*	0.94<br>*p<.001*	0.86<br>*p<.001*	0.99<br>*p<.001*
**NAR**	0.69<br>*p<.001*	0.88<br>*p<.001*	0.88<br>*p<.001*	0.90<br>*p<.001*	0.56<br>*p<.001*	0.90<br>*p<.001*	0.79<br>*p<.001*	0.95<br>*p<.001*	0.75<br>*p<.001*	0.90<br>*p<.001*	0.92<br>*p<.001*	0.87<br>*p<.001*	0.92<br>*p<.001*	0.92<br>*p<.001*	0.91<br>*p<.001*	0.81<br>*p<.001*	0.87<br>*p<.001*

Table S16. Additional descriptors correlated with polarity descriptors used in the paper (Pearson coefficient and p-values)

	nN	nO	nHet
**TPSA**	0.85<br>*p<.001*	0.86<br>*p<.001*	0.97<br>*p<.001*
**H-Acceptors**	0.89<br>*p<.001*	0.89<br>*p<.001*	0.98<br>*p<.001*
**H-Donors**	0.68<br>*p<.001*	0.69<br>*p<.001*	0.78<br>*p<.001*

Table S17. Additional descriptors which are not correlated with descriptors used in the paper (Pearson coefficient and p-values)

	nX	C%	N%	O%	X%	nStructures	nROR	Rbrid	MCD	RFD	RCI	RBF	NNRS	nR05
**MW**	0.47<br>*p<.001*	0.00<br>*7.80E-01*	0.08<br>8.20E-01	-0.24<br>*p<.001*	0.23<br>*p<.001*	0.00<br>*8.40E-01*	0.33<br>*p<.001*	0.42<br>*p<.001*	0.57<br>*p<.001*	0.30<br>*p<.001*	0.62<br>*p<.001*	0.60<br>*p<.001*	0.52<br>*p<.001*	0.69<br>*p<.001*
**nC**	0.40<br>*p<.001*	0.12<br>*p<.001*	0.04<br>*1.13E-02*	-0.28<br>*p<.001*	0.17<br>*p<.001*	-0.01<br>*6.28E-01*	0.30<br>*p<.001*	0.42<br>*p<.001*	0.58<br>*p<.001*	0.30<br>*p<.001*	0.61<br>*p<.001*	0.60<br>*p<.001*	0.51<br>*p<.001*	0.68<br>*p<.001*
**NAR**	0.33<br>*p<.001*	0.08<br>*1.64E-05*	0.21<br>*p<.001*	-0.39<br>*p<.001*	0.18<br>*p<.001*	-0.01<br>5.42E-01	0.16<br>*p<.001*	0.52<br>*p<.001*	0.72<br>*p<.001*	0.40<br>*p<.001*	0.69<br>*p<.001*	0.54<br>*p<.001*	0.53<br>*p<.001*	0.79<br>*p<.001*
**TPSA(Tot)**	0.35<br>*p<.001*	-0.17<br>*p<.001*	0.18<br>*p<.001*	-0.13<br>*p<.001*	0.14<br>*p<.001*	-0.01<br>*7.70E-01*	0.32<br>*p<.001*	0.44<br>*p<.001*	0.53<br>*p<.001*	0.33<br>*p<.001*	0.60<br>*p<.001*	0.60<br>*p<.001*	0.48<br>*p<.001*	0.74<br>*p<.001*
**H-Acceptors**	0.31<br>*p<.001*	-0.20<br>*p<.001*	0.23<br>*p<.001*	-0.09<br>*p<.001*	0.11<br>*p<.001*	0.00<br>7.81E-01	0.42<br>*p<.001*	0.48<br>*p<.001*	0.54<br>*p<.001*	0.36<br>*p<.001*	0.61<br>*p<.001*	0.52<br>*p<.001*	0.46<br>*p<.001*	0.71<br>*p<.001*
**H-Donors**	0.28<br>*p<.001*	-0.15<br>*p<.001*	0.17<br>*p<.001*	-0.12<br>*p<.001*	0.14<br>*p<.001*	0.00<br>8.67E-01	0.09<br>*p<.001*	0.29<br>*p<.001*	0.41<br>*p<.001*	0.23<br>*p<.001*	0.49<br>*p<.001*	0.67<br>*p<.001*	0.41<br>*p<.001*	0.57<br>*p<.001*
**PHI**	0.37<br>*p<.001*	-0.04<br>*3.77E-02*	-0.17<br>*p<.001*	0.07<br>*3.54E-05*	0.11<br>*p<.001*	0.00<br>*0.85*	0.58<br>*p<.001*	0.15<br>*p<.001*	0.16<br>*p<.001*	0.04<br>*1.79E-02*	0.28<br>*p<.001*	0.45<br>*p<.001*	0.29<br>*p<.001*	0.45<br>*p<.001*
